# Selective plasticity of layer 2/3 inputs onto distal forelimb controlling layer 5 corticospinal neurons with skilled grasp motor training

**DOI:** 10.1016/j.celrep.2024.113986

**Published:** 2024-04-09

**Authors:** Yoshio Takashima, Jeremy S. Biane, Mark H. Tuszynski

**Affiliations:** 1 Department of Neurosciences, UCSD, La Jolla, CA 92093, USA; 2 Department of Psychiatry, UCSF, San Francisco, CA 94158, USA; 3 VA Medical Center, San Diego, CA 92161, USA; 4 Lead contact

## Abstract

Layer 5 neurons of the neocortex receive their principal inputs from layer 2/3 neurons. We seek to identify the nature and extent of the plasticity of these projections with motor learning. Using optogenetic and viral intersectional tools to selectively stimulate distinct neuronal subsets in rat primary motor cortex, we simultaneously record from pairs of corticospinal neurons associated with distinct features of motor output control: distal forelimb vs. proximal forelimb. Activation of Channelrhodopsin2-expressing layer 2/3 afferents onto layer 5 in untrained animals produces greater monosynaptic excitation of neurons controlling the proximal forelimb. Following skilled grasp training, layer 2/3 inputs onto corticospinal neurons controlling the distal forelimb associated with skilled grasping become significantly stronger. Moreover, peak excitatory response amplitude nearly doubles while latency shortens, and excitatory-to-inhibitory latencies become significantly prolonged. These findings demonstrate distinct, highly segregated, and cell-specific plasticity of layer 2/3 projections during skilled grasp motor learning.

## In brief

Takashima et al. uncover how neurons in the motor cortex adapt during skilled forelimb grasp training. They reveal that layer 2/3 projections strengthen connections to distal forelimb control neurons, enhancing motor control. These findings illuminate cell-specific plasticity in motor learning, shedding light on brain mechanisms behind skilled motor learning.

## Graphical Abstract

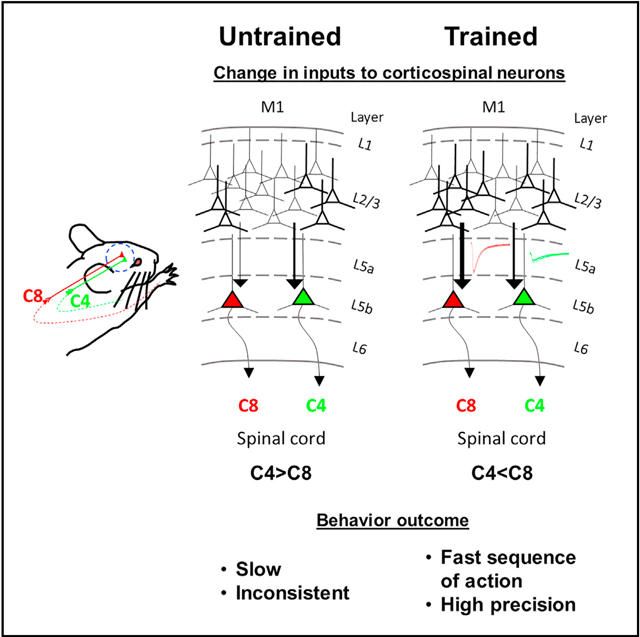

## INTRODUCTION

Motor learning represents the process of improvement in the precision and accuracy of trained movements over time. With motor learning, there is significant plasticity of motor cortical representational maps^1–3^ together with increases in spine number and the complexity of dendritic architecture in the motor cortex.^4–9^ Layer 5b corticospinal neurons dispatch the movement command directly to the spinal cord along with other indirect inputs. Recent studies have demonstrated significant plasticity associated with skilled forelimb grasp motor learning in the form of increased lateral connectivity among layer 5b corticospinal neurons that project to spinal cord segments controlling the forepaw.^10^ Furthermore, skilled grasp learning is associated with plasticity of thalamocortical inputs to layer 5b corticospinal neurons, limited once again to subsets of neurons projecting to spinal cord segments controlling distal musculature.^11^

Several neural systems that modulate motor control converge in the primary motor cortex (M1) to execute motor acts. These include associational inputs from the frontal cortex, parietal cortex, and contralateral motor cortex, as well as subcortical inputs arising from the thalamus.^12–17^ The most abundant input onto cortical layer 5 in all areas of the cortex arises from layer 2/3.^18–20^ Yet, plasticity of layer 2/3 onto layer 5 projections during motor learning in the adult brain has not, to date, been examined. Here, we subjected rats to skilled forelimb grasp training and used viral intersectional tools, *in utero* electroporation, optogenetics, and electrophysiological recordings to examine specific alterations in inputs from layer 2/3 onto layer 5b neurons among subsets of corticospinal neurons controlling either the distal or the proximal forelimb. We now find a highly significant strengthening of layer 2/3 inputs onto selective task-relevant layer 5b corticospinal neurons upon skilled motor learning together with significant increases in excitatory response amplitudes, reductions in excitatory response latencies, and prolongation in excitatory-to-inhibitory latencies. These plastic changes likely serve to refine a coordinated sequence of voluntary movements that are required to perform skilled motor tasks with speed, accuracy, and consistency.

## RESULTS

**Figure 1.**
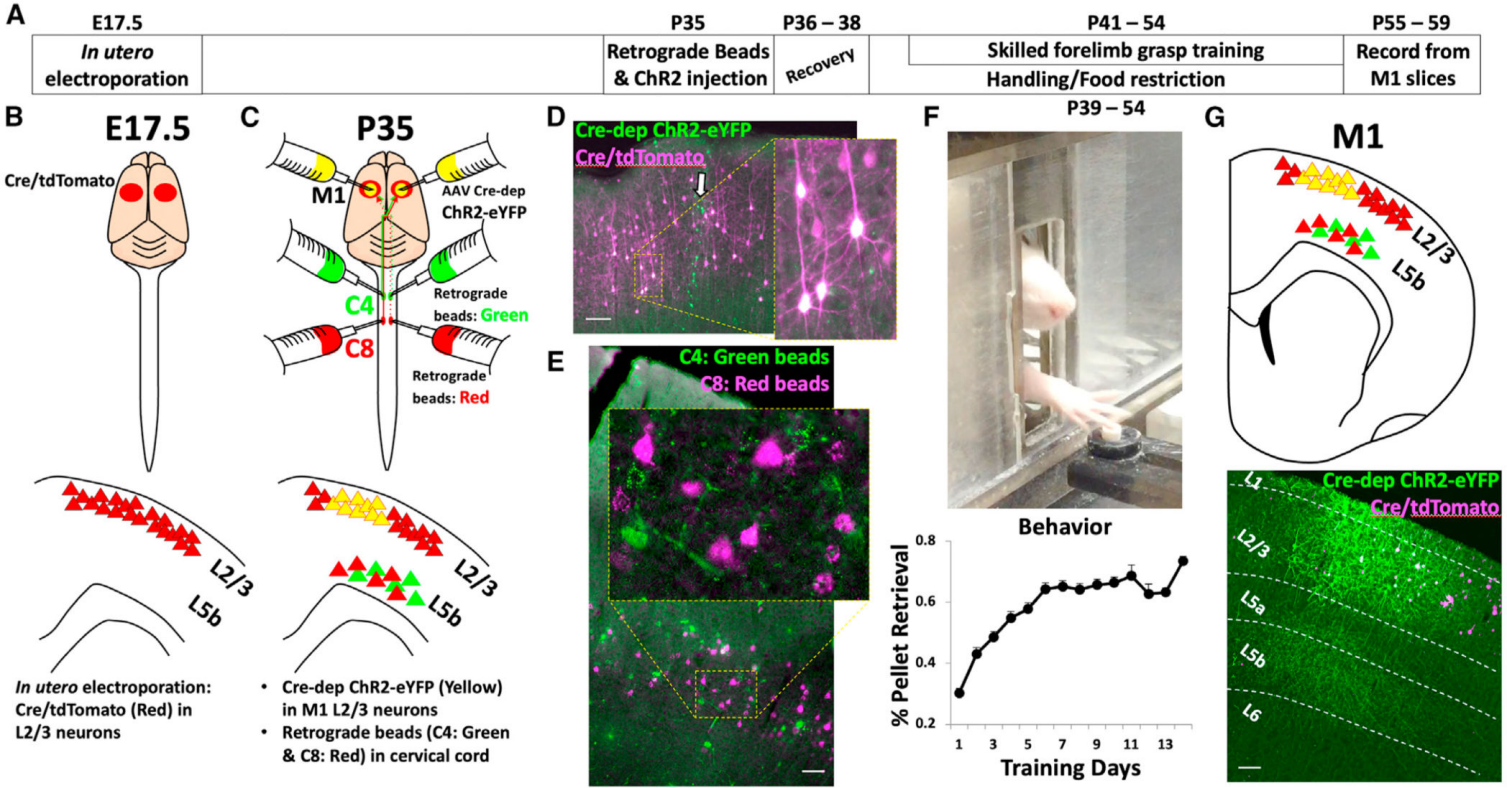
Experimental overview (A) Experimental timeline. (B) *In utero* electroporation was conducted at E17.5 to express Cre/tdTomato specifically in neocortical layer 2/3. (C) At P35, AAV 1/5 EF1α-DIO-hChR2(H134R)-EYFP was injected into caudal forelimb region of M1 to promote specific and restricted expression of ChR2-EYFP in M1 layer 2/3 neurons. Concurrently, two differently colored retrogradely transported Alexa-fluorophore-conjugated microspheres were injected at C4 (green) and C8 (red) of the spinal cord. These microspheres labeled distinct populations of corticospinal neurons controlling proximal- or distal-forelimb musculature, respectively, originating from layer 5b of the caudal M1. (D) Sample image of Cre/tdTomato (red) and ChR2-EYFP (green/white) expression in layer 2/3 of M1 cortical region of a slice. Arrow in white indicates needle track of AAV DIO-ChR2-EYFP injection. Shown is a region at higher magnification of layer 2/3 neurons expressing EYFP. (E) Sample image of Alexa-fluorophore-conjugated microspheres labeling somata of neurons in layer 5b of an M1 slice. Two differently colored fluorescence retrograde microsphere tracers were injected in C4 (green) or C8 (red) of the spinal cord, respectively, allowing separate assessment of these distinct neuronal populations in slices. (F) Skilled forelimb grasp training was performed over 14 consecutive days. Performance plateaued after 2 weeks. Repeated-measures ANOVA shows significant learning over time, **p < 0.001 (error bar: standard error). (G) Schematic representation of cortical slices used for electrophysiological recordings (top) and a sample image of ChR2 expression in M1 slice (bottom). ChR2-expressing layer 2/3 neurons extend axons to layer 5b (the location of corticospinal cell bodies) as indicated by robust EYFP expression. Scale bars: (D) 75 μm, (E) 50 μm, and (G) 75 μm.

To selectively excite caudal M1 layer 2/3 inputs onto layer 5, we used *in utero* electroporation to introduce Cre/tdTomato plasmids into the superficial M1 at embryonic day (E)17.5; *in utero* electroporation was performed at E17.5 because this day in neural development effectively targets layer 2/3 neurons in rat neocortex (Figures 1 and S1).^21^ We subsequently injected Cre-dependent AAV1/5-DIO channelrhodopsin2 yellow fluorescent protein (AAV1/5 EF1α-DIO-hChR2(H134R)-EYFP) into the same animals at postnatal day 35 (P35) targeting caudal M1; thus, following recombination, ChR2-EYFP was selectively expressed in caudal M1 layer 2/3 neurons and their axons in layer 5 (Figure 1). Labeling for cell-type-specific markers indicated that ChR2-EYFP was expressed nearly exclusively in neurons (Figure S1), rather than glia, and in excitatory neurons based on regular spiking patterns induced by depolarizing current injections (Figure S2D) and an absence of colocalization with inhibitory neuronal markers (Figures S2G–S2J).

As we reported previously, individual neurons of layer 5b in the caudal M1 that control the distal forelimb (i.e., wrist and digits) and are associated with performance of a skilled grasping behavior can be individually labeled in brain slices including the M1 by injection of retrogradely transported Alexa Fluor-conjugated microspheres at the cervical 8 (C8) spinal cord segment.^7^ Similarly, neurons controlling the proximal forelimb (i.e., shoulder and arm) can be individually labeled in the same slices by injecting a different colored set of retrogradely transported fluorescent microspheres into the C4 spinal cord segment.^7,22–24^ These C4- and C8-projecting neurons are intermingled in the M1 (Figure 1).^1,11^ These methods previously revealed that layer 5b neurons within the M1 exhibit increased spine density and dendritic complexity, increased lateral synaptic connectivity, and augmented excitation from thalamocortical inputs after skilled motor grasp training. These modifications were specifically restricted to only the C8-projecting corticospinal population, which is activated when performing a skilled grasping task^7,10,25^; in contrast, the C4-projecting corticospinal neurons in M1 layer 5b, which control proximal forelimb musculature, undergo no plastic changes. Using similar retrograde labeling techniques to selectively record responses from layer 5b corticospinal neurons following stimulation of layer 2/3 inputs, we injected differently colored Alexa-fluorophore-conjugated microspheres into either the C4 or C8 spinal cord segment. This resulted in retrograde transport of the fluorescent microspheres into somata of the C4- and C8-projecting layer 5b corticospinal neurons that were readily visualized in M1 slices (Figure 1).

**Figure 2.**
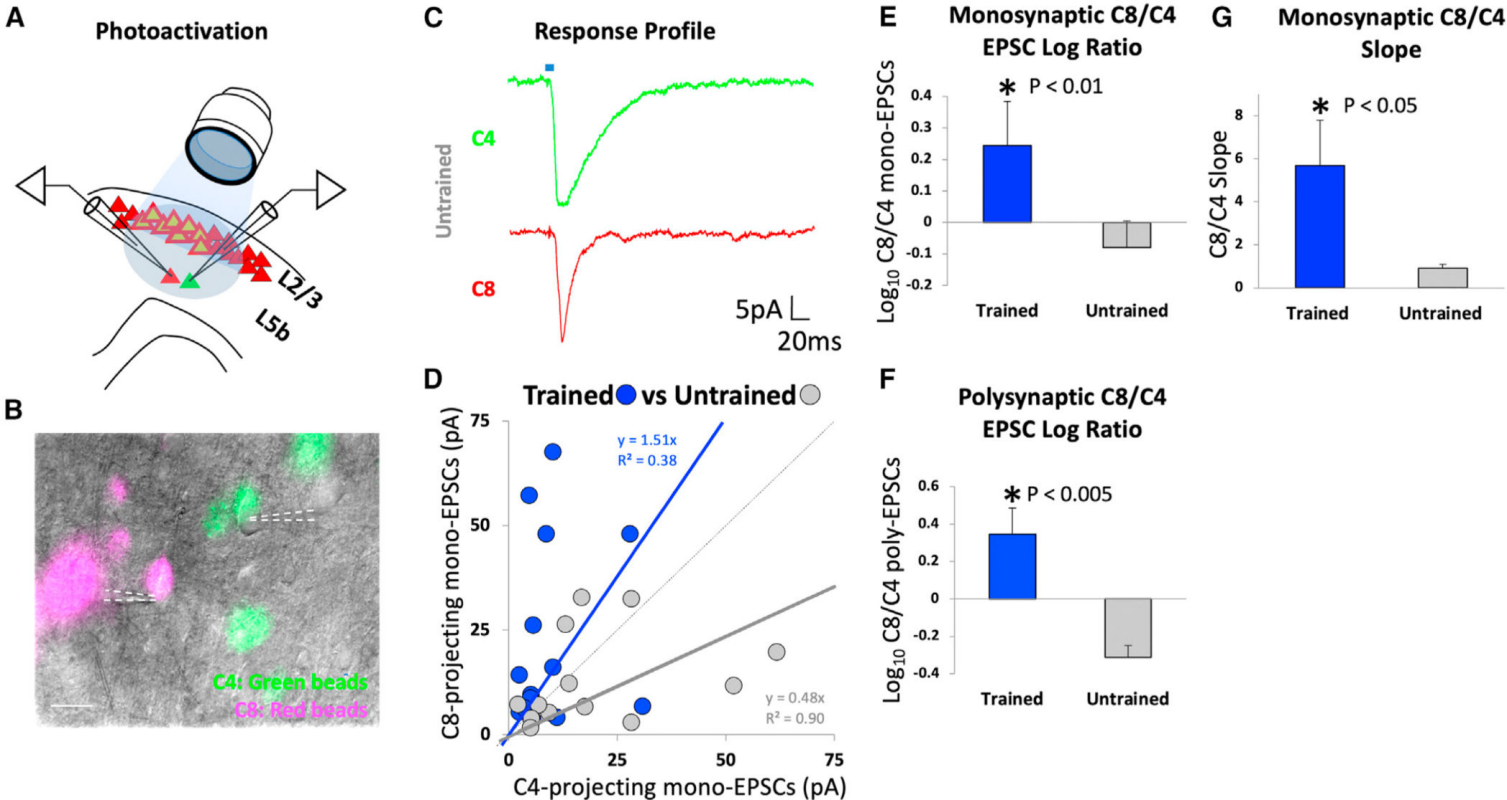
Optogenetic stimulation reveals stronger excitatory synaptic drive from layer 2/3 excitatory neurons onto layer 5b C8-projecting corticospinal neurons after skilled motor training (A) Schematic representation of whole-cell patch-clamp recording. Photo-stimulation at ~470 nm (5 ms) was applied under a 403 objective to selectively activate ChR2-expressing axonal terminals originating from caudal M1 layer 2/3 excitatory neurons (see Figure S2). Photo-induced EPSCs were recorded simultaneously from pairs of C4- (green) and C8-projecting corticospinal neurons (red) located in M1 layer 5b. (B) 40× image of simultaneously recorded pair of C4- and C8-projecting corticospinal neurons. Fluorescence images were overlaid on top of the bright-field image. (C) Representative sample traces of simultaneous whole-cell patch-clamp recording from pair of C4- and C8-projecting corticospinal neurons from untrained rats. (D) Relationship of evoked monosynaptic EPSCs in pairs of C4- and C8-projecting corticospinal neurons from skilled-forelimb-grasp-trained (n = 15; N = 8) vs. untrained rats (n = 14; N = 8). Analysis across all neuronal pairs indicates that skilled motor learning is associated with greater excitatory synaptic drive from layer 2/3 excitatory inputs onto layer 5b, C8-projecting neurons, evidenced by a shift in the ratio trend line (blue) toward the C8 population. (E) Log_10_ ratio of C8- to C4-projecting monosynaptic EPSC amplitudes. The ratio is significantly greater in skilled-forelimb-grasp-trained compared to untrained rats (*p < 0.01, two-tailed t test). (F) Log_10_ ratio of C8- to C4-projecting polysynaptic EPSC peak amplitudes. The ratio is significantly greater in skilled-forelimb-grasp-trained compared to untrained rats (*p < 0.005, two-tailed t test). (G) There is a significant shift in the C8/C4 slope comparing skilled-motor-trained and untrained rats (*p < 0.05, two-tailed t test comparing trained vs. untrained data points). All error bars represent standard errors. Scale bars: (B) 100 μm and (C) 50 μm.

After recovery from injections of tracers and viruses at P35, animals were divided into one of two groups: skilled forelimb grasp trained (N = 8) or untrained (N = 8); the latter group was handled and food restricted similarly to the trained group. Skilled forelimb grasp training resulted in an improvement in the accuracy of food retrieval from ~30% at the end of day 1 of training to ~70% on day 14, a statistically significant improvement from baseline performance (Figure 1F; *p < 0.05, paired two-tailed t test). 1–5 days following the completion of skilled motor training (P55–P59), acute cortical slices containing the caudal forelimb M1 region were prepared, and the C4- and C8-projecting corticospinal neurons near the center of the ChR2-expressing axon band in layer 5b were targeted for whole-cell patch-clamp recording (Figure 2). Activation of layer 2/3 terminals was achieved via blue LED light pulses (~470 nm, 5 ms) delivered through a 403 objective centered over the recorded cells in the M1 (Figures 2 and S3). Photo-induced excitatory postsynaptic currents (EPSCs) were recorded in a voltage-clamp configuration (holding cells at —70 mV) simultaneously between neuronal pairs of C8- and C4-projecting corticospinal neurons. The quality of whole-cell patch-clamp recordings was similar among all groups (Figure S4). Simultaneous paired recordings of C8- and C4-projecting corticospinal neurons were critical since small differences in the angle of the acute brain slices, health of the tissue, and irregularity in the number or intensity of ChR2 expression could potentially result in large slice-to-slice variations in response amplitude, making small differences difficult to detect. These potential confounds were addressed by making all comparisons as ratios of responses comparing between simultaneously recorded pairs of C8- and C4-projecting corticospinal neurons.

### Prior to motor learning, layer 2/3 inputs to layer 5 are stronger onto C4-projecting than C8-projecting neurons

We recorded monosynaptic and polysynaptic EPSCs in layer 5b corticospinal neurons resulting from photo-stimulation of layer 2/3 excitatory inputs in the M1. A total of 14 neuronal pairs were measured over 12 slices (N = 8). At baseline prior to skilled grasp training, stimulation of layer 2/3 inputs to layer 5b evoked stronger EPSCs in C4-projecting neurons than C8-projecting neurons (Figure 2D; *p < 0.05, Wilcoxon matched pairs signed rank test) as reflected by the slope of the evoked EPSC curve (Figure 2D, gray, y = 0.48) deviating markedly toward the C4 population in paired recordings.

### Skilled motor training biases layer 2/3 inputs toward C8-projecting neurons

We hypothesized that skilled motor learning would strengthen layer 2/3 inputs to layer 5 neurons that specifically control distal forelimb musculature, i.e., C8-projecting layer 5b corticospinal neurons. We recorded a total of 15 cell pairs over 13 slices (N = 8). Indeed, we found that skilled forelimb grasp training significantly increased the amplitude of EPSCs onto C8-projecting layer 5b corticospinal neurons compared to C4-projecting corticospinal neurons (Figure 2E). There was a significant shift in the slope of the evoked EPSC curve (Figure 2E, blue, y = 1.51) toward the C8 population in paired recordings (Figure 2G; *p < 0.05, two-tailed t test, comparing slopes of untrained vs. trained derived by taking the y/x ratio for each point and treating it as a distribution). Following training, the amplitude of monosynaptic responses in the grasp-related C8-projecting layer 5b neurons following light-evoked stimulation of layer 2/3 inputs was ~2.5-fold greater compared to the C4-projecting population that controls proximal forelimb movement (Figure 2E; *p < 0.01, two-tailed t test). This increase was not due to a reduction in layer 2/3 to C4-projection amplitude (Figure S3G). Moreover, comparison of the log10 ratio of polysynaptic C8/C4 EPSCs under trained vs. untrained conditions also demonstrated a significant shift toward the C8-projecting neuronal population (Figure 2F; *p < 0.005, two-tailed t test). These findings indicate that projections of layer 2/3 onto layer 5b exhibit substantial and significant plasticity in the context of adult skilled motor learning that is specific to the neurons mediating grasping that project to the C8 spinal cord.

**Figure 3.**
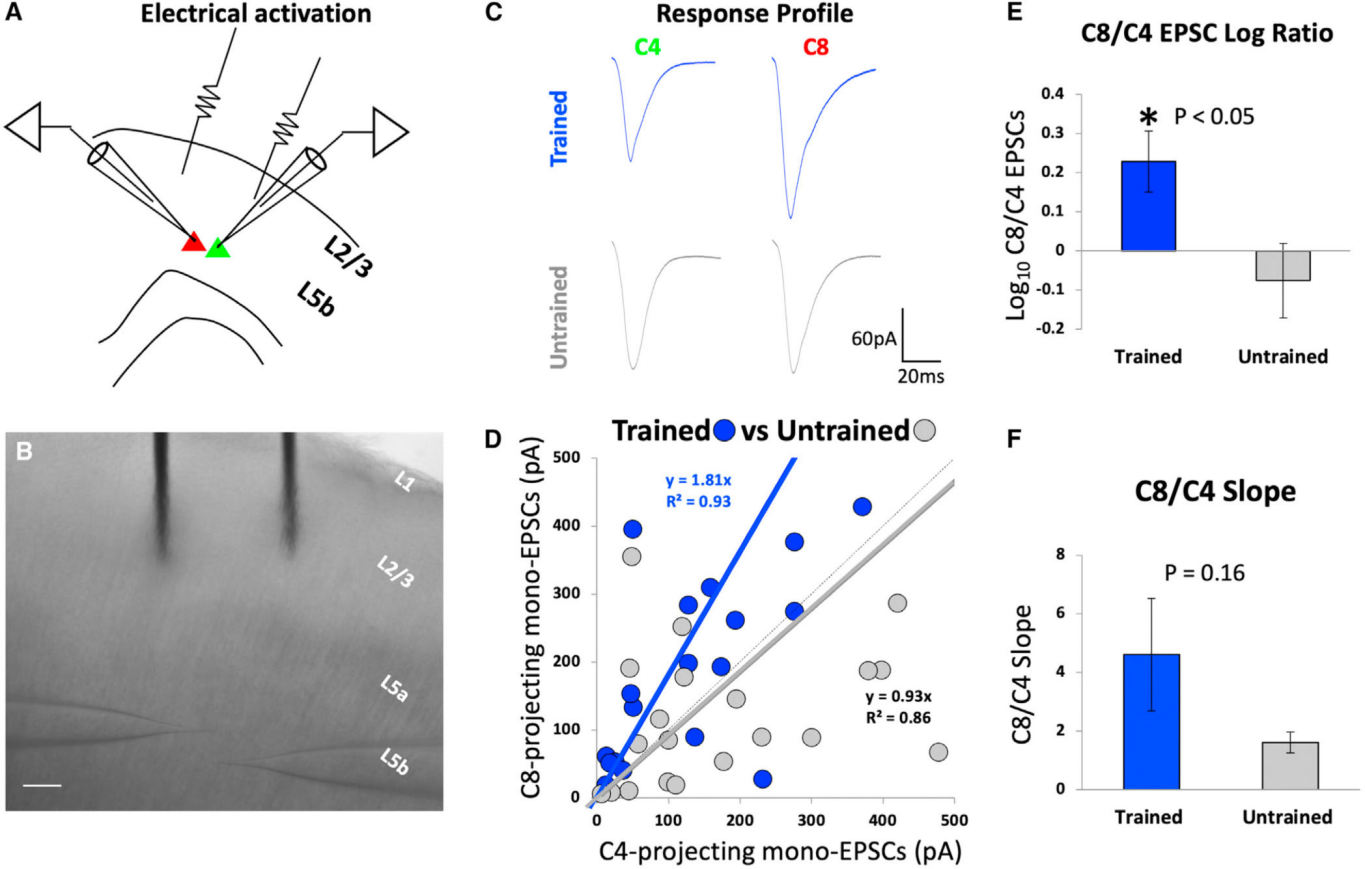
Electrical stimulation reveals greater excitatory synaptic drive from M1 layer 2/3 inputs onto layer 5b, C8-projecting corticospinal neurons after skilled motor training (A) Schematic representation of whole-cell patch-clamp recording. Layer 2/3 neurons were electrically stimulated, and evoked EPSCs were recorded simultaneously from pairs of C4- and C8-projecting corticospinal neurons in caudal M1 layer 5b. (B) 5× image of simultaneous paired recordings setup. Extracellular bipolar stimulus electrode was placed in layer 2/3 of M1 directly above the pairs of layer 5b, C4- and C8-projecting corticospinal neurons recorded. (C) Representative traces of simultaneous whole-cell patch clamp recordings from pair of C4- (Green) and C8-projecting (Red) corticospinal neurons from skilled motor trained and untrained rats. Stimulus artifact has been edited for clarity. (D) Relationship of evoked EPSCs in pairs of C4- and C8-projecting corticospinal neurons from skilled forelimb grasp trained (n = 21; N = 17) vs. untrained rats (n = 25; N = 15). Analysis across all neuronal pairs indicates that skilled motor learning is associated with a significant increase in excitatory synaptic drive from layer 2/3 inputs onto the layer 5b, C8-projecting population (blue line). In contrast, evoked EPSCs in untrained rats were slightly greater in the C4-projecting population (thick gray line; thin gray line is unity line). (E) Log_10_ ratio of C8- to C4-projecting EPSC peak amplitudes is significantly greater in skilled forelimb grasp trained compared to untrained rats (*p < 0.05, two-tailed t test). (F) There is a trend toward a higher C8/C4 slope among trained compared to untrained animals (p = 0.16, two-tailed t test). All error bars represent standard errors.

In addition to employing optogenetics to examine the projection from layer 2/3 to layer 5b plasticity in the context of skilled motor learning, in a separate set of 15 untrained and 17 trained animals, we employed direct electrical stimulation to confirm the results of optogenetic studies and to identify response properties of cells. As in optogenetic experiments above, acute cortical slices were prepared from both skilled-motor-trained (n = 21 pairs) and untrained rats (n = 25 pairs). Putative layer 2/3 neurons (both excitatory and inhibitory) were electrically stimulated using an extracellular bipolar stimulus electrode. Short-latency evoked EPSCs were recorded simultaneously from pairs of C8- and C4-projecting corticospinal neurons in voltage-clamp configuration holding the cells at —70 mV (Figure 3).

Among untrained animals, stimulation of layer 2/3 inputs to layer 5b evoked slightly greater EPSCs in C4-projecting corticospinal neurons compared to C8-projecting neurons as reflected by the slope of the evoked EPSC trend curve (Figure 3D, gray, y = 0.93). The magnitude of difference between the C4- and C8-projecting neurons differs from our optogenetic findings, where inputs were even stronger onto layer 5b, C4- than C8-projecting neurons. This difference between optogenetic and electrical stimulation data likely arises from the fact that electrical stimulation activates not only excitatory but also inhibitory neurons in layer 2/3. Moreover, electrical stimulation also potentially activates axons of passage within the electrical field and thalamocortical inputs. In contrast, optogenetic stimulation purely isolates responses arising from excitatory neurons in layer 2/3. Consistent with optogenetic stimulation data, the log10 ratio of C8/C4 EPSCs was ~2-fold higher after training than before (Figure 3E; *p < 0.05, two-tailed t test). Also consistent with optogenetic stimulation data, the mean slope of C8/C4 evoked EPSCs increased following skilled grasp training (Figure 3F; p = 0.16, two-tailed t test).

The post-training increase in amplitude of EPSCs in layer 5b, C8-projecting neurons evoked by layer 2/3 inputs could hypothetically result from increased presynaptic release probability. However, analysis of the pair-pulse ratio (PPR) did not support this interpretation: the PPR was similar among layer 5b, C4- and C8-projecting neurons in both trained and untrained conditions (Figure S5). It is also possible that plasticity occurs in the postsynaptic neurons as described in Biane et al.^11^ Furthermore, we have previously observed significantly increased numbers and densities of spines in C8-projecting corticospinal neurons after skilled grasp training suggesting that these neurons could receive a larger number of ascending inputs via layer 2/3.^7^

### Skilled motor training shortens the peak latency and duration of excitatory inputs to layer 5b and prolongs the latency and duration of inhibitory inputs

**Figure 4.**
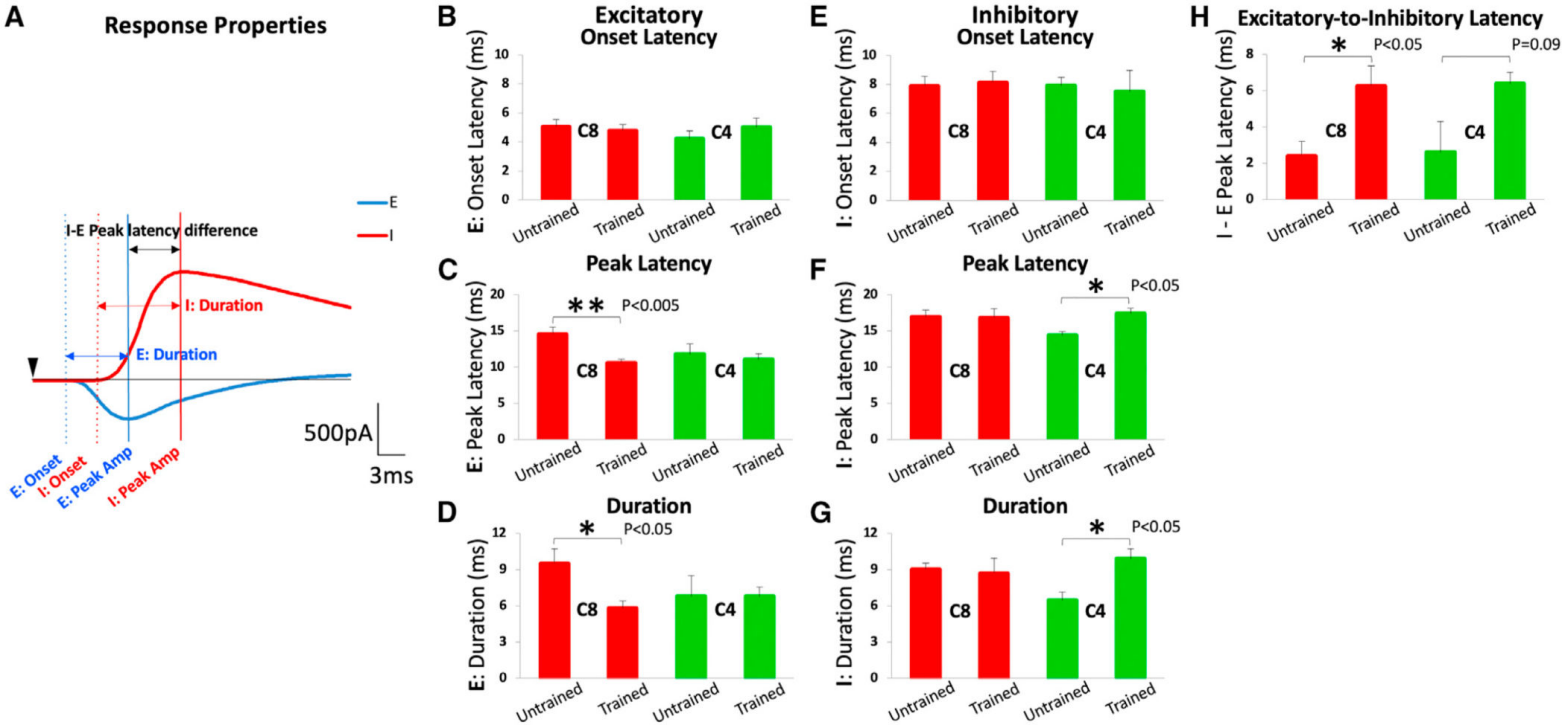
Patterns of excitatory responses from layer 2/3 inputs in layer 5b, C8- and C4-projecting corticospinal neurons after skilled motor training (A) Representative traces of evoked EPSCs and IPSCs elicited by M1 layer 2/3 electrical stimulation. EPSCs were recorded in voltage-clamp configuration by holding cells at —70 mV, whereas IPSCs were recorded by holding cells at +10 mV from the same C4- and C8-projecting corticospinal neurons located in M1 layer 5b (e.g., skilled-motor-trained rat). (B) Onset latencies of EPSCs did not differ significantly among groups. (C and D) Peak latency (C) and duration (D) of EPSCs were significantly shorter in C8-projecting neurons after skilled motor training (**p < 0.005 and *p < 0.05, respectively, two-tailed t test). (E) Onset latencies of IPSCs also did not differ significantly among C8- or C4-projecting neurons as a function of skilled motor grasp training. (F and G) Peak latency (F) and duration (G) of inhibitory responses became significantly prolonged among C4-projecting neurons after skilled motor training (*p < 0.05 and *p < 0.05, respectively, two-tailed t test) but did not change among C8-projecting neurons. (H) This resulted in significant prolongation in excitatory-to-inhibitory latencies in C8-projecting corticospinal neurons (*p < 0.05, two-tailed t test). All error bars represent standard errors.

We then tested the hypothesis that temporal and spatial summation of excitatory and inhibitory synaptic potentials arise from layer 2/3 neurons, which influence the generation of action potentials in layer 5b corticospinal neurons. In acute cortical slices containing the caudal M1, layer 2/3 was electrically stimulated, and evoked EPSCs or inhibitory postsynaptic currents (IPSCs) were recorded among both C8- and C4- projecting corticospinal neurons from skilled-motor-trained and untrained rats (Figure 4A). Cells were held in voltage clamp at either —70 or +10 mV for EPSCs and IPSCs, respectively, from the same cell. Following stimulation of layer 2/3 inputs, the onset latency of excitatory and inhibitory responses did not differ significantly among trained and untrained animals in C8- and C4-projecting neurons (Figures 4B and 4E). In contrast, the time to peak excitatory latency (measured from time of stimulation to time of peak amplitude) was significantly reduced in rats after skilled motor training compared to untrained rats in the C8-projecting population but not in the C4-projecting population (Figure 4C; **p < 0.005, two-tailed t test). Furthermore, the duration of the EPSC was reduced following training only among C8-projecting neurons (Figure 4D; *p < 0.05, two-tailed t test).

Notably, the peak latency and duration of IPSCs were significantly longer after training in C4-projecting, but not in C8-projecting, corticospinal neurons (Figures 4F, *p < 0.05, and 4G, *p < 0.05, respectively, two-tailed t test). Thus, skilled motor training shortened the peak latency and duration of excitatory input from layer 2/3 onto layer 5b, C8-projecting corticospinal neurons controlling the distal forelimb (i.e., wrist and digits). At the same time, inhibitory inputs onto layer 5b, C4-projecting corticospinal neurons controlling the proximal forelimb musculature (i.e., shoulder and arm) became prolonged. This resulted in significant prolongation in excitatory-to-inhibitory latencies in C8-projecting corticospinal neurons (Figure 4H; *p < 0.05, two-tailed t test).

### Ablation of layer 2/3 excitatory inputs impairs skilled forelimb grasping accuracy

The preceding results indicate that inputs from layer 2/3 onto layer 5b undergo substantial plasticity during skilled motor learning and that the preponderance of these changes are focused on the C8-projecting population of layer 5b corticospinal neurons that control the distal forelimb to execute skilled grasping. To assess whether these plastic changes are in fact required for rats to execute their newly learned skilled motor behavior, we used an intersectional viral approach to selectively ablate layer 2/3 excitatory neurons upon completion of motor learning.

**Figure 5.**
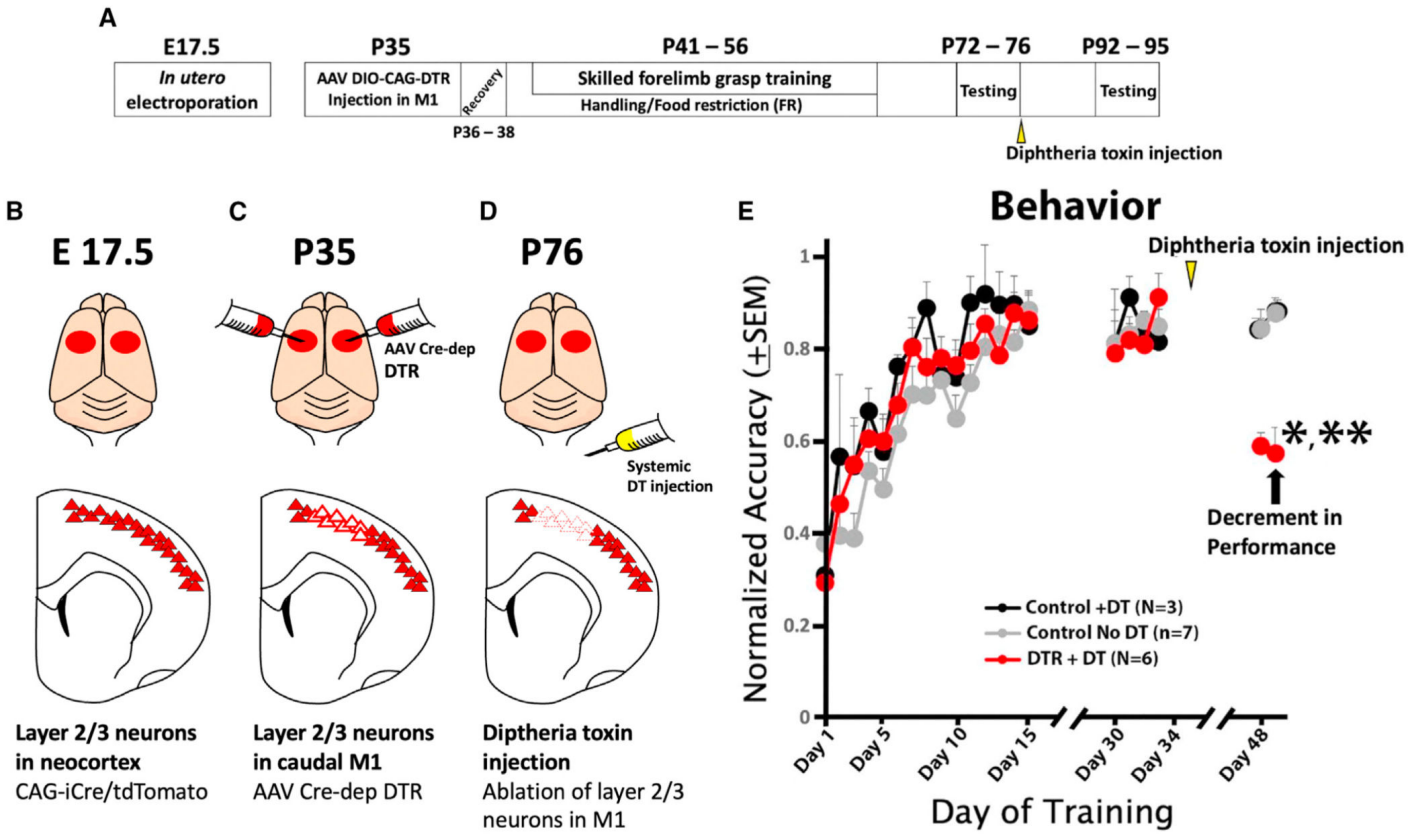
Layer 2/3 excitatory neurons are essential for skilled forelimb grasping performance accuracy (A) Experimental timeline (B) *In utero* electroporation was performed at E17.5 to express Cre/tdTomato specifically in neocortical layers 2/3. (C) At P35, AAV9 FLEX-DTR was injected into caudal M1 to promote specific and restricted expression of DTR in M1 layer 2/3 excitatory neurons. (D) At P76, after completion of skilled forelimb grasp training, DT was injected, ablating layer 2/3 excitatory neurons in M1. (E) Skilled forelimb grasp training was performed, demonstrating significant improvement in pellet retrieval accuracy to >80% (***p < 0.001, repeated-measures ANOVA). Rats underwent repeated testing 2 weeks later to ensure persistence of retrieval ability, followed by intraperitoneal injections of either DT in 6 animals that expressed the DTR and 3 controls that did not or saline in 7 controls that expressed DTR. DT injections are indicated by yellow arrowheads. Two weeks later, only animals expressing DTR that received DT injections demonstrated a significant reduction in grasping performance (***p < 0.001, ANOVA). **p < 0.005 indicates significant loss of grasping accuracy among animals with DTR + DT injections after DT injections compared to both control groups. *p < 0.05 indicates significant difference in pre-DT performance compared to post-DT performance in the DTR + DT group. The averages of days 32–35 and 48–49 were compared for statistical analysis. All error bars represent standard errors.

First, we utilized *in utero* electroporation to express Cre/tdTomato plasmids in layer 2/3 of the M1 at E17.5 under the CAG promoter (Figures 5A and 5B). At P35, we injected Cre-dependent human diphtheria toxin receptor (DTR) using an AAV9 FLEX-DTR into the M1 (N = 13 rats); 3 control rats underwent sham surgery. All animals then underwent skilled forelimb grasp training, reaching >80% proficiency by day 15. At the end of the training period prior to receiving DT, the trained groups exhibited equal grasping proficiency (Figure 5E; p = 0.8, two-tailed t test). At the end of skilled motor testing, 6 animals that had been intra- cortically injected with AAV9 FLEX-DTR received subcutaneous injections of DT to ablate layer 2/3 excitatory neurons, 7 animals that had been intracortically injected with AAV9 FLEX-DTR received injections of saline (rather than DT) as controls, and 3 animals that underwent sham surgery and did not receive intracortical injections of AAV9 FLEX-DTR injections received DT also as controls (Figures 5C–5E). Repeat skilled forelimb grasp testing 2 weeks later showed a significant, 42% reduction in performance of skilled forelimb grasping only among animals that received injections of AAV9 FLEX-DTR followed by subcutaneous DT injections; the two control groups showed no decrement at all (Figure 5E; *p < 0.05, post hoc paired two-tailed t test of AAV9 FLEX-DTR + DT before vs. after performance; p = 0.23, paired two-tailed t test of AAV9 FLEX-DTR + saline control group yielded no significant difference; p = 0.97, paired two-tailed t test of control AAV9 FLEX-DTR + saline control group yielded no significant difference). The absolute performance of animals that received AAV9 FLEX-DTR + DT was also significantly worse than the two control groups (Figure 5E; **p < 0.005, two-tailed t test of AAV9 FLEX-DTR + DT vs. AAV9 FLEX-DTR + saline; **p < 0.005, two-tailed t test, AAV9 FLEX-DTR + DT vs. sham surgery + DT). This magnitude of drop is noteworthy given that AAV9 FLEX-DTR injections likely infected less than the majority of layer 2/3 neurons projecting to layer 5b. Subsequent immunolabeling of sections demonstrated an expected loss of neurons in layer 2/3 neurons in regions where AAV9 FLEX-DTR was injected followed by DT administration (Figure S6). We also tested overground locomotion, an unskilled behavior, on the CatWalk in all groups. DT injections did not significantly perturb forelimb-hindlimb coordination (Figure S6). These results demonstrate that the layer 2/3 motor cortex is required for accuracy on the skilled forelimb grasping task.

## DISCUSSION

Findings of this study demonstrate that the principal inputs to layer 5b cortical output neurons, layer 2/3 projections, undergo extensive plasticity with motor learning in the adult brain. The strength of excitatory inputs from layer 2/3 onto layer 5b increases by more than 2-fold as an adult rat undergoes skilled motor grasp training, and the peak latency of excitatory response significantly shortens, together with the duration of the response. Strikingly, nearly all of these changes are restricted to the subpopulation of corticospinal output neurons in layer 5b that project to the distal forelimb control region of the spinal cord (C8) and command the wrist and digits executing the skilled grasping behavior. The same measures in layer 5b neurons projecting to the shoulder and arm region, C4-projecting corticospinal neurons, are static, other than a prolongation of peak latency and duration of inhibitory response properties. As shown in previous work, C8-projecting layer 5b neurons subserve skilled grasping behavior and exhibit a significant increase in spine numbers and dendritic complexity with skilled grasp training.^7^ Thus, the changes in layer 2/3 projections onto layer 5b are narrowly parcellated onto subsets of learning-relevant neurons. This spatiotemporal orchestration of behaviorally relevant subpopulations of corticospinal neurons likely allows the animal to perform a skilled motor task with speed, accuracy, and consistency.

Motor learning has served as a highly tractable model system for understanding plasticity in the adult, learning brain. Early and mid-stages of motor learning are dependent on the M1 and are associated with increased numbers, turnover and stabilization of selective synapses,^9,26^ newly acquired spatiotemporal sequences of neural activity^6,27^ and alterations in multiple dynamic representations.^26,28–32^ Later-stage motor learning and performance becomes more independent of the M1.^33^ During the M1-dependent stage of early- and mid-stage learning, we and others have sought to identify features of neural plasticity that underlie the spatiotemporal sequences and dynamic representations brought about by learning. Indeed, multiple levels of synaptic plasticity converge onto layer 5b corticospinal neurons projecting to C8 during the learning of a skilled motor grasping task. First, “learning” layer 5b neurons projecting to spinal level C8 exhibit a significant, 22% expansion in spine number together with more elaborate apical and basilar dendritic branches with learning.^7^ Second, layer 5b neurons projecting to spinal level C8 exhibit a ~2.5-fold increase in lateral synaptic connectivity and increased excitability with other C8-projecting neurons, but not with C4-projecting layer 5b neurons, upon learning the skilled grasping task.^10^ Third, thalamic inputs to the M1 exhibit a significant, ~2-fold increase in input strength onto the layer 5b, C8-projecting neurons with skilled grasp learning but not with layer 5b, C4-projecting neurons.^11^ Fourth, skilled forelimb grasp training is associated with long-term potentiation in M1.^34^

The present study now adds to this knowledge by demonstrating that the most abundant inputs to layer 5 neurons—layer 2/3 inputs^18,19,21^—are also strengthened during skilled grasp learning. These changes consist of strengthened and shorter latency excitatory inputs from layer 2/3 onto layer 5b corticospinal neurons projecting to C8 together with prolongation of inhibitory inputs to layer 5b, C4-projecting corticospinal neurons. Thus, the orchestration of synaptic plasticity is extensive and recruited from multiple inputs yet narrowly focused onto task-relevant neuronal subpopulations, consistent with previous observations of narrow parcellation of plastic changes onto behaviorally relevant subsets of neurons.^11,35^

The importance of the layer 5b, C8-projecting corticospinal population for skilled motor performance has been confirmed by silencing of this subset of neurons using intersectional viral approaches to express inhibitory DREADDs^36^; this resulted in significant impairment in skilled grasping performance (Figure 4 in Biane et al.^10^). In the present study, we further confirm that the layer 2/3 neurons are also critical for skilled forelimb grasping behavior using intersectional viral techniques combined with *in utero* electroporation. The ablation of layer 2/3 neurons affected not only layer 2/3-to-5b projections but all projections arising from layer 2/3. Thus, our results demonstrate that the layer 2/3 motor cortex is required for accuracy on the skilled forelimb grasping task.

### Limitations of the study

The role of layer 2/3 / layer 5b inputs in this study was established using both optogenetic approaches, which allowed specific activation of excitatory inputs from layer 2/3 to layer 5b, and traditional electrical stimulation studies employing simultaneous paired recordings in M1 slices using patch-clamp methods. Both techniques were quite consistent in demonstrating a significant enhancement of excitatory inputs to layer 5b neurons with skilled motor learning. Yet, the optogenetic approach is likely the more specific approach of the two because it allowed selective activation of excitatory inputs exclusively arising from layer 2/3 by virtue of heavily biased viral infection of excitatory neurons. Traditional electrophysiological approaches may also activate axons of passage and thalamocortical inputs, likely accounting for the higher amplitude responses observed with direct stimulation.

## STAR★METHODS

### RESOURCE AVAILABILITY

#### Lead contact

Further information and requests for resources and reagents should be directed to and will be fulfilled by the lead contact, Yoshio Takashima (yoshi.takashima@gmail.com).

#### Materials availability

This study did not generate new unique reagents. There are no restrictions for use of the material disclosed.

#### Data and code availability

- All data reported in this paper will be shared by the lead contact upon request.
- This paper does not report original code.
- Any additional information required to reanalyze the data reported in this paper is available from the lead contact upon request.

### EXPERIMENTAL MODEL AND STUDY PARTICIPANT DETAILS

All experimental procedures and animal care adhered to American Association for the Accreditation of Laboratory Animal Care and University of California, San Diego/Veterans Affairs Medical Center, San Diego guidelines for experimental animal health, safety, and comfort.

A total of 99 Fischer 344 male rats were subjects of this study, 16 of which were included for optogenetic stimulation experiments (age: P55–59), 67 for electrical stimulation experiments (age: P55–59), and 16 for ablation of M1, layer 2/3 neurons behavioral experiments (age: P95). Animals were group housed (2–3/cage) in standard laboratory cages on a 12hrs light/dark cycle. Each cage was enriched with a cardboard tube.

### METHOD DETAILS

#### *In utero* electroporation

Timed pregnant rats (E17.5) were anesthetized with a drug cocktail (2 mL/kg) containing ketamine, xylazine, and acepromazine (25 mg/ml, 1.3 mg/ml, 0.25 mg/ml; respectively). An incision was made in the abdominal midline and all uterine horns were extracted onto a 37°C pre-warmed phosphate-buffered saline (PBS) moistened cotton gauze, which was placed around the wound area. Transillumination against the uterine wall was used to visualize embryos. To electroporate DNA, a glass micropipette with beveled tip (15^0^) was carefully inserted into the ventricle of the embryos and approximately 1 μg/μl of DNA constructs (CAG-Cre/tdTomato) were injected mixed with 0.5% Fast Green (Sigma-Aldrich) to both brain hemispheres. To target electroporation to superficial layers of the neocortex, the electrodes (Tweezertrodes; BTX Harvard Apparatus) were placed outside of the uterus near the cortex and square-wave current pulses (50mV, 5 pulses, 50ms with 1s interval) were applied to the embryo using ECM830 Electro Square Porator (BTX Harvard Apparatus). Post electroporation, the uterine horns were returned to the abdomen. Appropriate post-operative analgesics were given.

#### Neuronal retrograde labeling with Alexa-fluorophore conjugated latex microspheres

Male F344 rats at postnatal day P35 (weighing ~90g) were anesthetized with a drug cocktail (2 mL/kg) containing ketamine, xylazine, and acepromazine (25 mg/ml, 1.3 mg/ml, 0.25 mg/ml; respectively). In rats, ventral motor neuronal populations located in the cervical 4 (C4) spinal cord segment project to musculature of the proximal forelimb including the neck, shoulder, and arm. The C8 spinal cord segment contains ventral motor neuron pools that are associated with controlling the distal forelimb, including the forearm, wrist, and digits which are required for grasping movements.^7,10,11,22–24^ To label corticospinal neurons projecting to either the C4 or C8 segments of the spinal cord, laminectomies were performed and a glass micropipette (tip <40mm) containing red or green Alexa-fluorophore conjugated latex microspheres (RetroBeads; Lumafluor) were inserted through the dorsal part of spinal cord into the spinal cord at 0.7 and 1.0mm in depths and 0.55mm from midline). Different colored fluorophores were injected into C4 or C8 to allow subsequent segregation of these two populations in subsequent slices (Note: C4 is represented as Green and C8 as Red in order to simplify throughout the paper). 0.1mL of fluorescent latex microsphere beads were injected into 3 sites at 2 depths per side of the spinal cord. In all cases, tracer diffusion was assessed postmortem in 40mm coronal slices of the spinal cord to ensure accuracy of injections. Approximately 25%, of cortical layer 5b neurons expressed both fluorophores^25^ and these double-labeled neurons were not used.

#### AAV1/5 EF1α-DIO-hChR2(H134R)-eYFP injections

In the same surgical session that retrograde tracers were injected into the spinal cord, AAV1/5 EF1α-DIO-hChR2(H134R)-eYFP (1.0×10^12^; Penn/Addgene) was injected into the caudal forelimb region of the M1 in both brain hemispheres at the following injection sites: 1.0–0.0 A/P, 2.5–3.5 M/L, 0.7 and 1.1 D/V; A/P and M/L relative to bregma, D/V relative to pia. Injections were made using a glass micropipette and an infusion pump (Chemyx Inc.) delivering 0.1 μL at each site at a rate of 0.12 μL/min. Following infusion, the micropipette remained in place for 2min to allow adequate diffusion to the surrounding tissue. Among animals that received injections of Cre at E17.5, Cre recombined with DIO-ChR2-eYFP, restricting ChR2-eYFP expression to layer 2/3 neurons in caudal M1.

#### Skilled forelimb grasp training

At postnatal day P41, animals began skilled forelimb grasp training as described previously.^1,10,11,38,39^ Briefly, rats were first acclimated to an experimenter and a testing chamber for two days prior to initiation of grasping. Food restriction was maintained for the duration of the training to no less than 90% of age appropriate body weight. For training, rats reached through a small aperture in a Plexiglass chamber to grasp a single 45mg sucrose pellet (Test Diets) located on an indented platform approximately 2cm beyond the reaching chamber. Skilled forelimb grasp training was carried out across 14 consecutive days and rats performed approximately 60 trials or 15min of grasping per day. A trial was defined as an extension of the forepaw (reaching movement) beyond the chamber facade toward the pellet-containing platform followed by successful retrieval (grasping movement) of a sucrose pellet and consumption of the pellet.^40^ Skilled forelimb grasping performance accuracy was scored as the total number of successful trials divided by the total number of trials. To control for potential effects of food restriction, handling, or exposure to a novel food (reward sucrose pellets), control animals were also food restricted, handled, spent an equal amount of time (15min) in the testing chamber, and consumed approximately equal numbers of reward pellets as trained animals. However, controls were manually fed reward pellets with forceps, thus not allowing the animal to reach for or grasp reward pellets.

#### Acute brain slice preparation

Following completion of training, rats were very deeply anesthetized with a higher dose of the drug cocktail stated above and were perfused for 3min with ice-cold, oxygenated, 876 modified sucrose ACSF containing (in mM) 75 NaCl, 2.5 KCl, 3.3 MgSO4, 0.5 CaCl2, 1NaH2PO4, 26.2 NaHCO3, 22 glucose, 52.6 sucrose, 10 HEPES, 10 choline chloride, 1 pyruvate, 1 L-ascorbic acid (~300 mOsm, pH 7.4). The rat was rapidly decapitated and the brain was removed from the skull. 330mm coronal cortical slices including caudal M1 were cut at an angle 15^0^ anterior to the mid-coronal plane to match the apical dendritic projection pattern of layer 5b corticospinal neurons. Acute cortical slices were cut and collected in ice-cold, oxygenated, modified sucrose ACSF. Slices were transferred to an interface chamber containing the same oxygenated, modified sucrose ACSF solution and incubated at 34°C for 30min. Slices were then allowed to recover at room temperature (24°C) for additional 40min before initiating recordings. Recordings were carried out at room temperature in a submersion type recording chamber perfused with oxygenated ACSF containing (in mM) 119 NaCl, 2.5 KCl, 1.3 MgCl2, 2.5 CaCl2, 1.3 NaH2PO4, 26.0 NaHCO3, 20 glucose (~295 mOsm, pH 7.4) at a rate of 2–3 mL/min. Postnatal age at time of recording was between 55 and 59 days, an age where the motor system has fully matured.^10,11,25^

#### Electrophysiology

All recordings were performed with the cortical hemisphere contralateral to the preferred grasping paw. In the case of untrained animals, the hemisphere recorded was randomly selected. The experimenter conducting whole-cell patch clamp recordings was blinded to experimental group assignment of the animal. Neurons were selected based on emission spectra (red or green, reflecting tracers injected at either the C4 or C8 spinal segment; the experimenter was blind to the identity of the back-labeled cells) and then visualized under infrared differential interference contrast video microscopy (Olympus BX-51 scope and Rolera XR digital camera) for targeted whole-cell patch clamp recordings. Whole-cell voltage and current clamp recordings were made at room temperature using patch pipettes (4–7 MU) pulled with Model PC-10 (Narishige) filled with internal solution containing (in mM) 150 K-Gluconate, 1.5 MgCl2, 5.0 HEPES, 1 EGTA, 10 phosphocreatine, 2.0 ATP, and 0.3 GTP. No drugs were applied in the bath solution unless indicated in the text. Postsynaptic data were analyzed exclusively from cells with a resting membrane potential % −55mV, drift less than 6mV over the entire recording period, access resistance %35MU, an ability to evoke multiple spikes with >60mV peak amplitude from threshold, and with a holding current of > −500pA to keep the cell at a “native” resting membrane potential of —70mV. Basic membrane properties are reported only for cells whose holding current was >−150pA. Series resistance was not compensated, but was continuously monitored via negative voltage steps. Whole-cell patch clamp recordings were obtained using Multiclamp 700B patch amplifiers (Molecular Devices) and data analyzed using pClamp10 software (Molecular Devices). Data were low-pass filtered at 2kHz, and digitized at 10kHz.

#### Evoked excitatory postsynaptic currents

To record layer 2/3 evoked excitatory postsynaptic currents (EPSCs), neurons were recorded in voltage clamp configuration holding cells at —70mV. For whole-cell voltage-clamp recordings, the same internal electrode solution was used as stated above. Upon achieving the whole-cell configuration in a pair of labeled C4- and C8-projecting corticospinal neurons simultaneously, responses were evoked by either photo-stimulation (for optogenetic experiments) or extracellular electrical stimulation in layer 2/3 just above the patched pair of neurons at a frequency of 0.1Hz (for electrical stimulation experiments). Note that use of the term “pair” does not indicate that the two neurons simultaneously recorded were synaptically connected. To isolate evoked EPSCs, neurons were held at —70mV and responses were collected. Layer 2/3 stimulation intensity was gradually increased until a short-latency (3–6 ms) EPSC was observed in both recorded neurons. Instances in which one or both of the neurons failed to respond were not included in analysis, and subsequently another pair was attempted. For most pairs in which reliable responses could be evoked, EPSCs were then collected at multiple stimulation intensities to sample a range of response amplitudes <100pA for optogenetics experiments and typically ranging <500pA for electrical stimulation experiments. 10 to 20 stimulation trials were collected at each intensity and used for data analysis. To directly compare EPSCs recorded in different pairs and from different slice preparations, we analyzed evoked responses from trials taken at the minimum stimulation intensity capable of generating a reliable response in both neurons.

#### Optogenetic stimulation

Optogenetic blue light was delivered through a 403 objective with blue excitation irradiance maintained between 2 and 5 mW/mm^2^ (range of intensity at tissue), creating an area of light stimulation that spanned 100s of mm and uniform activation of ChR2-expressing axon terminals across simultaneously patched neighboring cells in the M1, layer 5b. Light emission originated from blue (470nm, 750mW) mounted LEDs (Thorlabs), 1200mA LED driver, FITC excitation filter (Chroma). Stimulation was controlled with a MultiCamp 700B patch-clamp amplifier via a Digidata 1440A digitizer using Clampex software (all from Molecular Devices). Photo-induced EPSCs were abolished in the presence of tetrodotoxin (TTX, 250nM; TOCRIS 1078) and 6,7-dinitroquinoxaline-2,3-dione (DNQX, 100mM; TOCRIS 0189).

#### Electrical stimulation

An extracellular bipolar stimulation electrode was placed in layer 2/3 of M1, directly above the pairs of layer 5b, C4- and C8-projecting corticospinal neurons (Figures 3A and 3B). To remove the possibility of prolonged stimulation altering experimental outcomes via plasticity induction, the stimulus electrode was moved to a new location within the same slice, or to a new slice after successful recording from a pair of neurons.

#### Evoked inhibitory postsynaptic currents

To record layer 2/3 evoked IPSCs, neurons were recorded in voltage clamped configuration holding cells at +10mV. IPSCs were evoked by extracellular electrical stimulation in layer 2/3 just above the patched pair of neurons at a frequency of 0.1Hz. The same stimulation intensities used to collect EPSCs were used for IPSCs from the same neurons. Instances in which one or both of the neurons failed to respond were not included in analyses and subsequently another pair was attempted. Response latencies for polysynaptic IPSCs were more variable than for EPSCs, and also changed sharply with stimulus intensity, likely due to the scattered distribution of inhibitory neurons in the vicinity of the stimulating or recording electrode. 10 to 20 stimulation trials were averaged at multiple stimulus intensities for each recorded pair of neurons.

#### Histology

Animals were anesthetized as above and transcardially perfused with 250mL cold phosphate buffered saline (0.1M, pH 7.4), followed by 250mL of cold 4% paraformaldehyde in 0.1M phosphate buffer. Brains were extracted, post fixed overnight in the same fixative, then cryoprotected in 0.1M phosphate buffer containing 30% sucrose for at least 72 h at 4°C. Sections were cut on a sliding microtome set to 40mm and stored in cryoprotectant (TCS) at 4°C until processed for immunohistochemistry. For all immunohistochemistry, free-floating staining methods were used. Coronal cortical sections were washed in TBS (x3, 15min each) and permeabilized with 0.25% TritionX-100 (30min). Non-specific labeling was blocked with 5% donkey serum in TBS (1hr). Sections were incubated overnight at room temperature in appropriate primary antibodies: RFP (Abcam, ab34771); GFP (Aves, GFP-1020); NeuN (Millipore, abn90); Calretinin (Encor bio, CPCA-Calretinin); Calbindin (Swant, cb300); GABA (Millipore, AB5016); Parvalbumin (Swant, pv235) diluted 1:1000 in TBS, 0.25% TritionX-100, and 5% donkey serum. Following primary antibody incubation, sections were wash in TBS (x3, 5min each) and incubated in appropriate Alexa-Fluor secondary antibodies (Invitrogen) diluted 1:300 in TBS for overnight at room temperature. Sections were again washed (x3, 5min each), mounted on glass slides and cover slipped. Images are obtained using Keyence microscope (Keyence) and processed with Fiji (ImageJ).

#### Diphtheria toxin injections

All animals were littermates. *In utero* electroporation (see above for more detail) was performed at E17.5 to express CAG-Cre/tdTomato plasmids in neocortical layer 2/3 neurons. At birth, pups were checked for Cre/tdTomato expression to ensure that the expression was located in M1 in both hemispheres. At P35, AAV9-bpAM-Cbh-FLEX human diphtheria toxin receptor-GFP-WPRE (AAV9 FLEX-DTR, 1.0×10^12^; Vigene Biosciences) was injected into caudal M1 (N = 13 rats) in both brain hemispheres while 3 controls underwent sham surgery. All animals were subsequently trained in skilled forelimb grasping and were retrained after a two week of gap to confirm retention of ability to perform the task with high performance accuracy. Then 9 animals received subcutaneous injection of diphtheria toxin (DT, 50 ng/g; Sigma-Aldrich), 6 DTR+ to eliminate layer 2/3 excitatory neurons in the M1 region, while 7 controls expressing AAV9 FLEX-DTR received injections of saline. Skilled forelimb grasping was assessed two weeks later.

### QUANTIFICATION AND STATISTICAL ANALYSIS

In this study, 99 male Fischer 344 rats were used as subjects. The breakdown of the experimental groups and ages is as follows: 16 rats for optogenetic stimulation experiments (age: P55–59), 67 rats for electrical stimulation experiments (age: P55–59), and 16 rats for DT ablation of M1, layer 2/3 neurons behavioral experiments (age: P95). Among these rats, 59 were trained for skilled forelimb grasp training, while 40 served as untrained controls.

For the optogenetic stimulation experiments, 8 rats were trained and 8 were untrained. In the electrical stimulation experiments,^41^ rats were trained and 32 were untrained. All 16 rats used for DT ablation were trained for skilled forelimb grasp training.

The statistical analyses were conducted using Microsoft Excel. The significance level was defined as follows: *p < 0.05, **p < 0.01,***p > 0.005, ***p < 0.001.

Statistical tests used in the study include.

1. Repeated measures ANOVA to demonstrate significant learning over time in skilled forelimb grasp training (Figures 1F; 5E).
2. Two-tailed paired t-tests to compare differences between specific days of training and pre-vs. post-treatment within and between groups (Figures 1F; 5E).
3. Wilcoxon matched pairs signed rank test for comparison between C4- and C8-projecting neurons at baseline (Figure 2D).
4. Two-tailed t-tests to compare means between trained and untrained groups (Figures 2E, 2F, 2G, 3E, 3F, 4B, 4C, 4D, 4F, 4G, 4H, 5E, S3E, S4A–S4E, and S5B).
5. Post-hoc paired two-tailed t-tests for specific comparisons after treatment (Figure 5E).
6. two-way ANOVA to compare intrinsic spiking between C4- and C8-projecting corticospinal neurons from skilled motor trained vs. untrained rats (Figure S4F).
7. Note: Two-tailed t-tests = unequal variances t-tests.

The statistical details of experiments, including the specific tests used, the number of subjects (N) or pairs recorded simultaneously (n), definition of center (mean), and dispersion and precision measures (standard error), can be found in the figure legends and results sections corresponding to each figure.

Strategies for randomization and/or stratification, sample size estimation, and inclusion and exclusion criteria for data or subjects are explicitly mentioned in the Method Details section. There were no methods to determine whether the data met assumptions of statistical approaches. Overall, the study employed a variety of statistical methods to analyze the data obtained from different experimental setups, and the specific details of these analyses are provided in the figure legends and results sections.

## Supplementary Material

1

## Figures and Tables

**Table:** KEY RESOURCES TABLE

REAGENT or RESOURCE	SOURCE	IDENTIFIER
Antibodies		

RFP	Abcam	Cat# AB34771; RRID: AB_777699
GFP	Antibodies Incorporated	Cat# GFP-1020; RRID: AB_10000240
NeuN	Millipore	Cat# ABN90; RRID: AB_11205592
Calretinin	Encor Biotechnology	Cat# CPCA-Calretinin; RRID: AB_2572241
Calbindin	Swant	Cat# 300; RRID: AB_10000347
GABA	Millipore	Cat# AB5016; RRID: AB_91634
Parvalbumin	Swant	Cat# 235; RRID: AB_10000343
Alexa-Fluor secondary antibodies	ThermoFisher Scientific (Invitrogen)	https://www.thermofisher.com/antibody/secondary/query/alexa

Bacterial and virus strains		

AAV 1/5 EF1α-DIO-hChR2(H134R)-eYFP-WPRE-HGHpA	Penn/Addgene	20298-AAV1 20298-AAV5
AAV9-bpAM-Cbh-FLEX human diphtheria toxin receptor-GFP-WPRE	Charles River (Prev. Vigene Biosciences)	N/A

Chemicals, peptides, and recombinant proteins		

Tetrodotoxin (TTX)	TOCRIS	Cat. No. 1078
6,7-dinitroquinoxaline-2,3-dione (DNQX)	TOCRIS	Cat. No. 0189
Diphtheria Toxin	Sigma-Aldrich	D0564

Experimental models: Organisms/strains		

Rat: Fischer 344	Envigo	N/A

Recombinant DNA		

CAG-Cre/tdTomato plasmid	This paper	N/A
AAV9-bpAM-Cbh-FLEX human diphtheria toxin receptor-GFP-WPRE (FLEX-DTR plasmid)	Azim et al., 2014^37^	N/A

Software and algorithms		

pClamp10 (Data analysis)	Molecular Devices	N/A
Fiji	ImageJ	https://fiji.sc

## Highlights

- Layer 2/3 neurons in the motor cortex adapt during skilled forelimb grasp training
- Plasticity results in stronger connections to distal forelimb control neurons
- Excitatory response doubles and peak latency shortens
- Findings reveal cell-specific plasticity in motor learning
