# Supplementary material for: Selective plasticity of layer 2/3 inputs onto distal forelimb controlling layer 5 corticospinal neurons with skilled grasp motor training
Source: Cell Rep. Author manuscript; Available in PMC 2025 Oct 16. (PMC12529104; doi:10.1016/j.celrep.2024.113986)

Cell Reports, Volume *43*

Supplemental information

Selective plasticity of layer 2/3 inputs onto distal forelimb controlling layer 5 corticospinal neurons with skilled grasp motor training

Yoshio Takashima, Jeremy S. Biane, and Mark H. Tuszynski

SUPPLEMENTARY FIGURE LEGENDS

Supplementary Figure 1: Specific Expression of Cre in Layer2/3 Neurons in Rat Neocortex

(A) A schematic of neocortical Cre/tdTomato expression after *in utero* electroporation at E17.5. (B) Sample whole brain image of corresponding Cre/tdTomato expression in cortical region including M1 after sacrifice at P56. (C) 10x image of 40µm-thick cortical slice in coronal section containing M1. (D) Higher magnification of panel C, showing exclusive tdTomato expression in layer2/3 neurons. (E) Cre/tdTomato is exclusively expressed in neurons, shown by colabeling with the neuron-specific marker, NeuN. Cre/tdTomato is not expressed in all the layer2/3 neurons within a region (see inset). Scale bars: B, 2.5mm; C,1.0mm; E, 40µm.

Supplementary Figure 2: Specific and Restricted Expression of ChR2-eYFP in M1, Layer2/3 Excitatory Neurons

(A) Schematic representation of whole-cell patch clamp recording of layer2/3 neurons expressing ChR2-eYFP. Photo-stimulation at ~470nm (5ms) applied across the cortical slice viewed at 40x magnification to selectively stimulate neurons expressing ChR2-eYFP in M1 layer2/3. Photo-induced responses were recorded from those neurons. (B) 5x bright field image of whole-cell patch clamp recording setup. (C) 40x image of whole-cell patch clamp recording ChR2-eYFP expressing layer2/3 neuron. Fluorescent image was overlaid on a bright field image. (D) Representative traces of action potentials elicited by depolarizing current injections. There is a regular spiking pattern suggesting that ChR2-expressing layer2/3 neurons are excitatory (n=10). (E) Representative traces of evoked spiking in M1, layer2/3 ChR-eYFP expressing neurons. Traces were obtained using whole-cell patch clamp recording in current clamp configuration. 5ms of photo-stimulation evokes a spike. (F) Pair pulse photo-stimulation (two 5ms stimulation with 50ms interval) evoked two action potentials. (G-J) Inhibitory neurons in M1 layer2/3 do not express ChR2-eYFP, shown by absence of colocalization with Calretinin, Calbindin, GABA, and Parvalbumin. Scale bars: B, 100µm; C, 50µm; G-J, 50µm.

Supplementary Figure 3: Layer5b Corticospinal Neurons Receive Direct Mono-synaptic Inputs from Layer2/3 Excitatory Neurons in M1

(A) Schematic representation of whole-cell patch clamp recording technique. Photo-stimulation at ~470nm (5ms) was applied under 40x magnification to selectively activate ChR2-expressing axonal terminals originating from caudal M1, layer2/3 excitatory neurons. Photo-induced EPSCs were recorded from Alexa-fluorophore conjugated microspheres labeled corticospinal neurons. (B) 5x image of ChR2-eYFP in layer2/3 (arrowhead); microspheres injected into spinal cord level C4 are retrogradely transported and label layer5b corticospinal neurons (arrow). Fluorescence image is overlaid on top of bright field image. (C) Higher magnification (40x) shows targeted whole-cell patch clamp in cortical slice that includes M1 region. A layer5b corticospinal neuron projecting to the C4 spinal cord segment is visible. Fluorescence image is overlaid on top of bright field image. (D) In the same field as panel C, eYFP labeled axons from layer2/3 excitatory neurons expressing ChR2 are visible (disambiguation of the eYFP signal from the Alexa 488-fluorophore conjugated microspheres was achieved via a narrow-band GFP filter). (E) Average amplitude from C4-projecting corticospinal neurons from trained vs untrained animals. There is a nonsignificant increase rather than reduction in amplitude from C4-projecting neurons after training. (F) Representative EPSC traces of whole-cell patch recording (voltage clamp configuration, holding cells at -70mV). For simplicity, one recording from a C4-projecting corticospinal neuron (green) is shown [individual sweep (10 sweeps in total) in black and an averaged sweep in red]. Stimulus onset is indicated by blue bar (5ms). (G) Light gray line shows abolition of photo-induced averaged EPSCs in presence of tetrodotoxin (TTX, 250nM). (H) Photo-induced averaged EPSCs were also abolished in the presence of the AMPA antagonist, 6,7-dinitroquinoxaline-2,3-dione (DNQX, 100uM). Scale bars: B, 100µm; C, 50µm; D, 50µm.

Supplementary Figure 4: No Change in Intrinsic Membrane Properties of C8- and C4-Projecting Corticospinal Neurons After Skilled-Motor Training

(A) Membrane resistance (Rm), (B) spike threshold, (C) resting membrane potential, (D) half-width of a spike and (E) rheobase (the minimum current that will produce an action potential)

did not differ significantly among groups. (F) C8-projecting corticospinal neurons from skilled-motor trained rats (n=30; N=24) show greater spiking frequency to 500ms of incrementally increased depolarizing current pulses compared to C8-projecting corticospinal neurons from untrained rats (n=44; N=26) (two-way ANOVA; *p<0.05). The current pulse-spike relationship did not differ between C4-projecting corticospinal neurons from skilled-motor trained (n=28; N=20) vs untrained rats (n=24; N=18) (two-way ANOVA; p=0.6).

Supplementary Figure 5: No Change in Pre-Synaptic Release Probability of Layer2/3 Inputs onto C8- and C4-Projecting Corticospinal Neurons After Skilled-Motor Training

Pre-synaptic release probability was measured by comparing ratio of response amplitudes of layer5b corticospinal neurons to two rapid activation pulses applied to the pre-synaptic population. (A) Sample traces are shown from untrained rats. Arrowhead indicates stimulus onset. Stimulus artifact has been truncated for clarity. (B) There is no change in PP2/PP1 among all cell types.

Supplementary Figure 6: Diphtheria Toxin Ablates M1, Layer2/3 Neurons in Rats that Express Diphtheria Toxin Receptor

(A) Ablation of layer2/3 neurons with DT did not result in perturbation of forelimb-hindlimb coordination during locomotion on the CatWalk. (B) Animals received *in utero* electroporation of CAG-Cre/tdTomato plasmids at E17.5 to specifically express Cre in neocortical layer2/3 excitatory neurons, followed by injections of AAV9 FLEX-DTR into caudal M1 at P35. Following behavioral training and retraining, some rats received intraperitoneal injection of DT resulting in ablation of layer2/3 neurons. (C-D) Higher magnification of right and left hemispheres, respectively, showing loss of layer2/3 neurons expressing Cre/tdTomato at sites of DTR injection in the M1 region. Scale bar: B,100µm.

Supplementary Figure 1

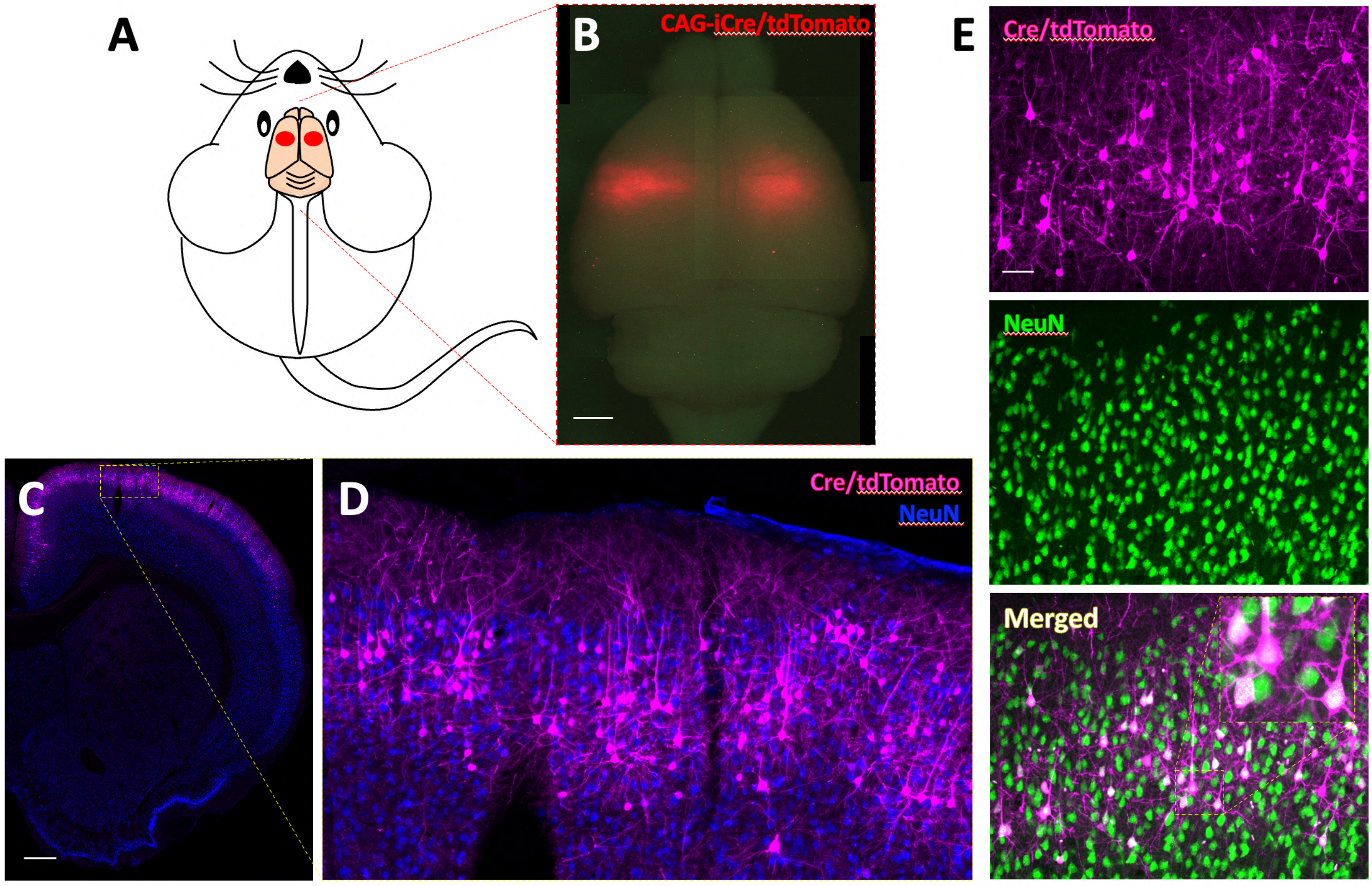

Supplementary Figure 2

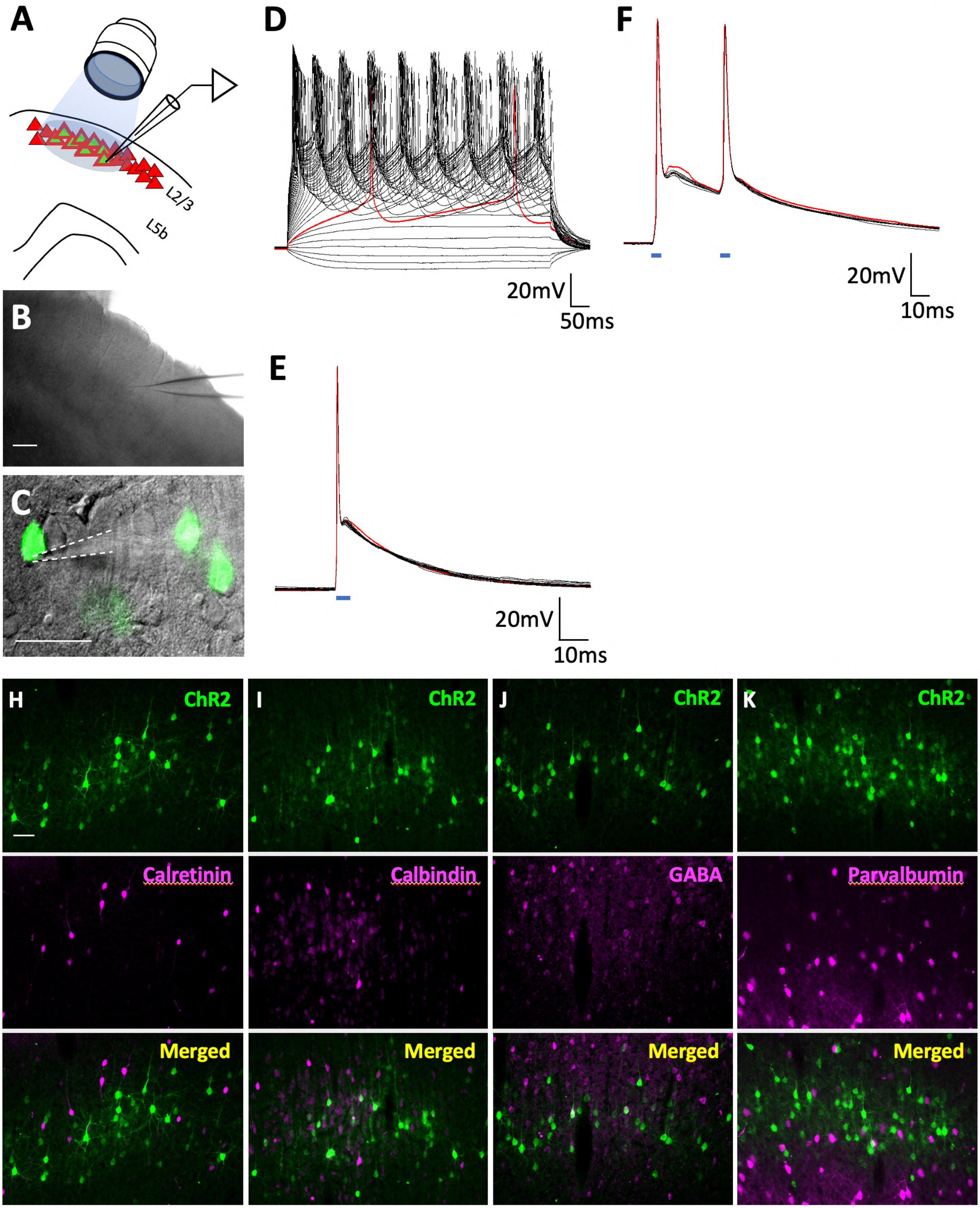

Supplementary Figure 3

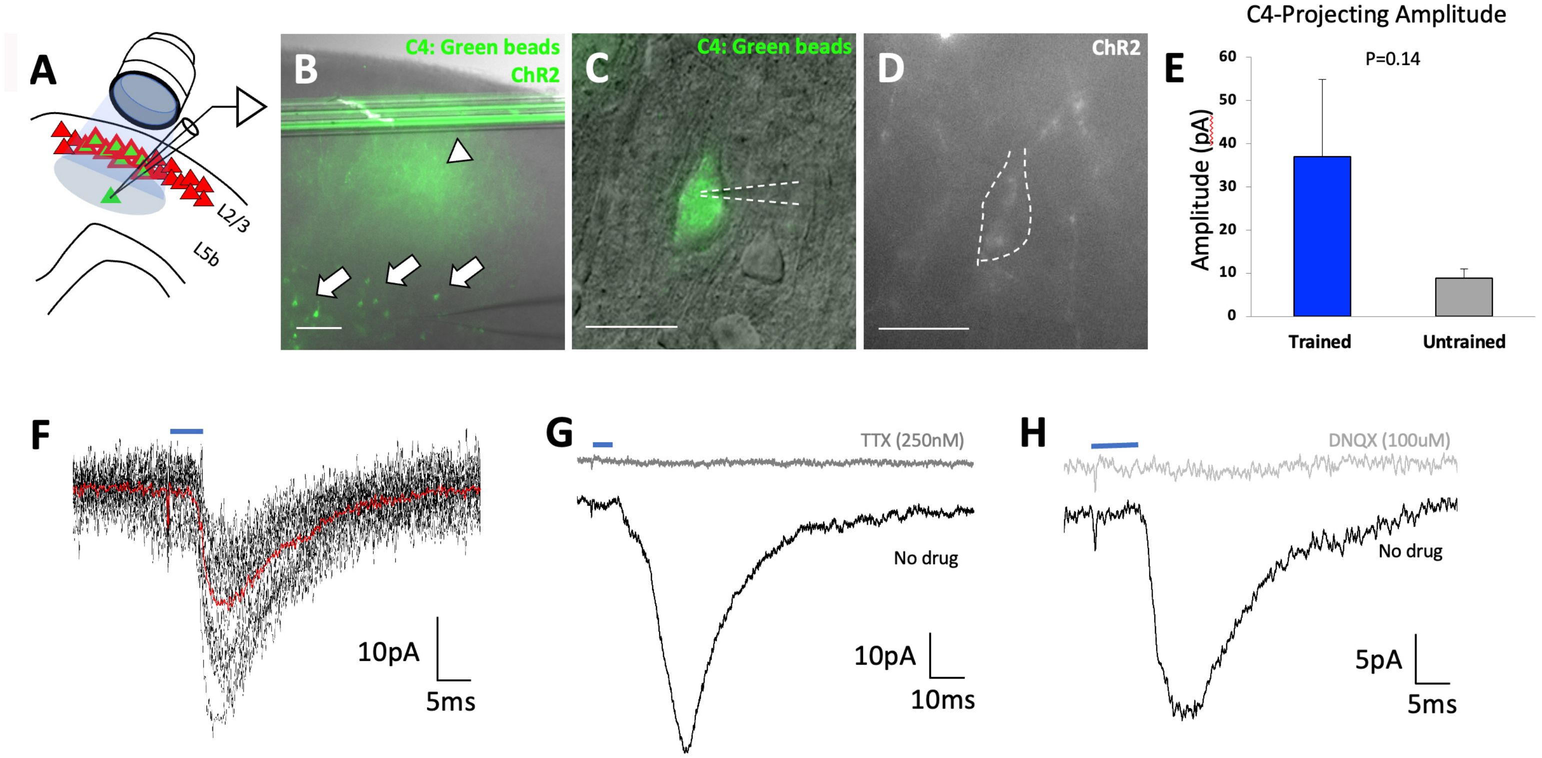

Supplementary Figure 4

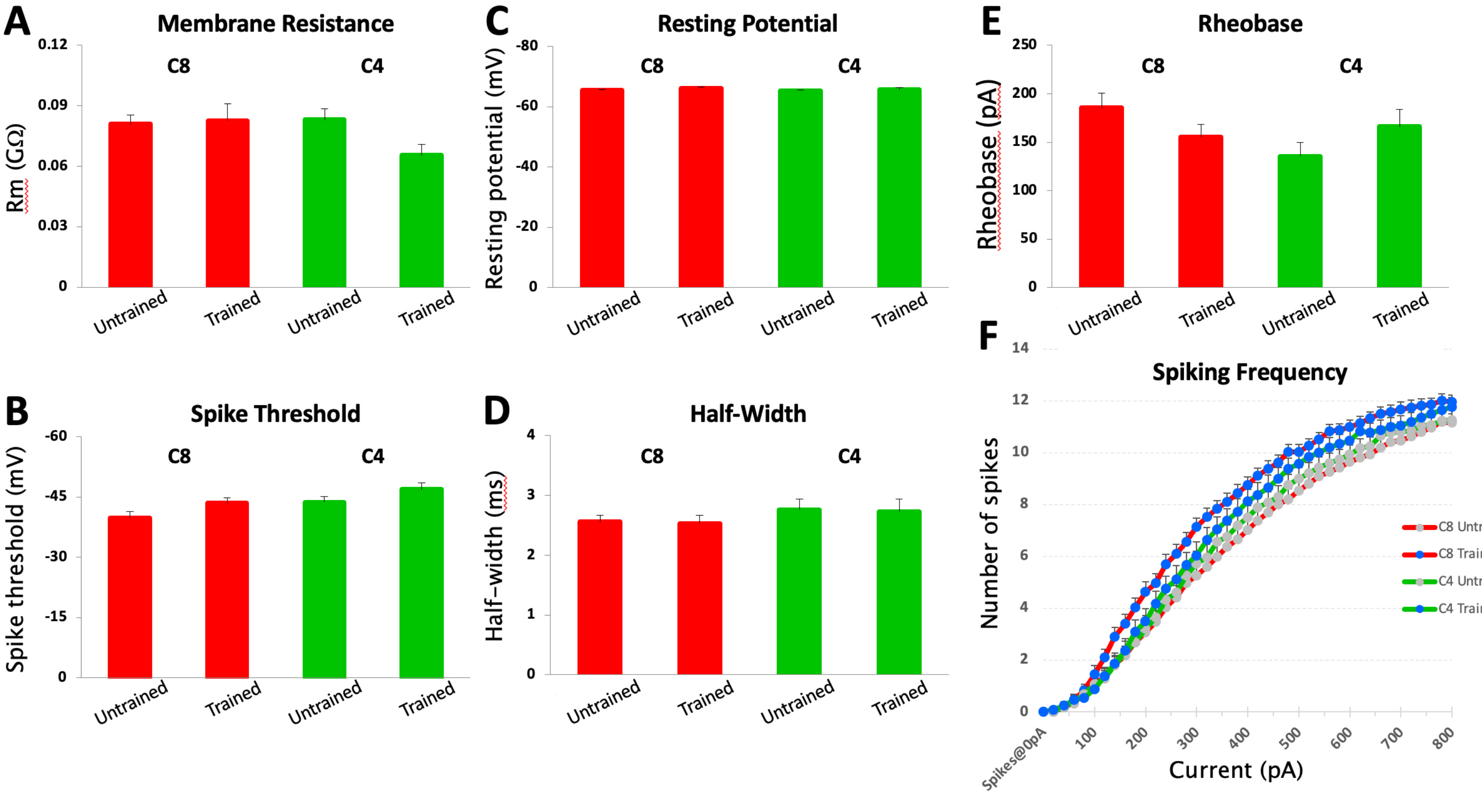

Supplementary Figure 5

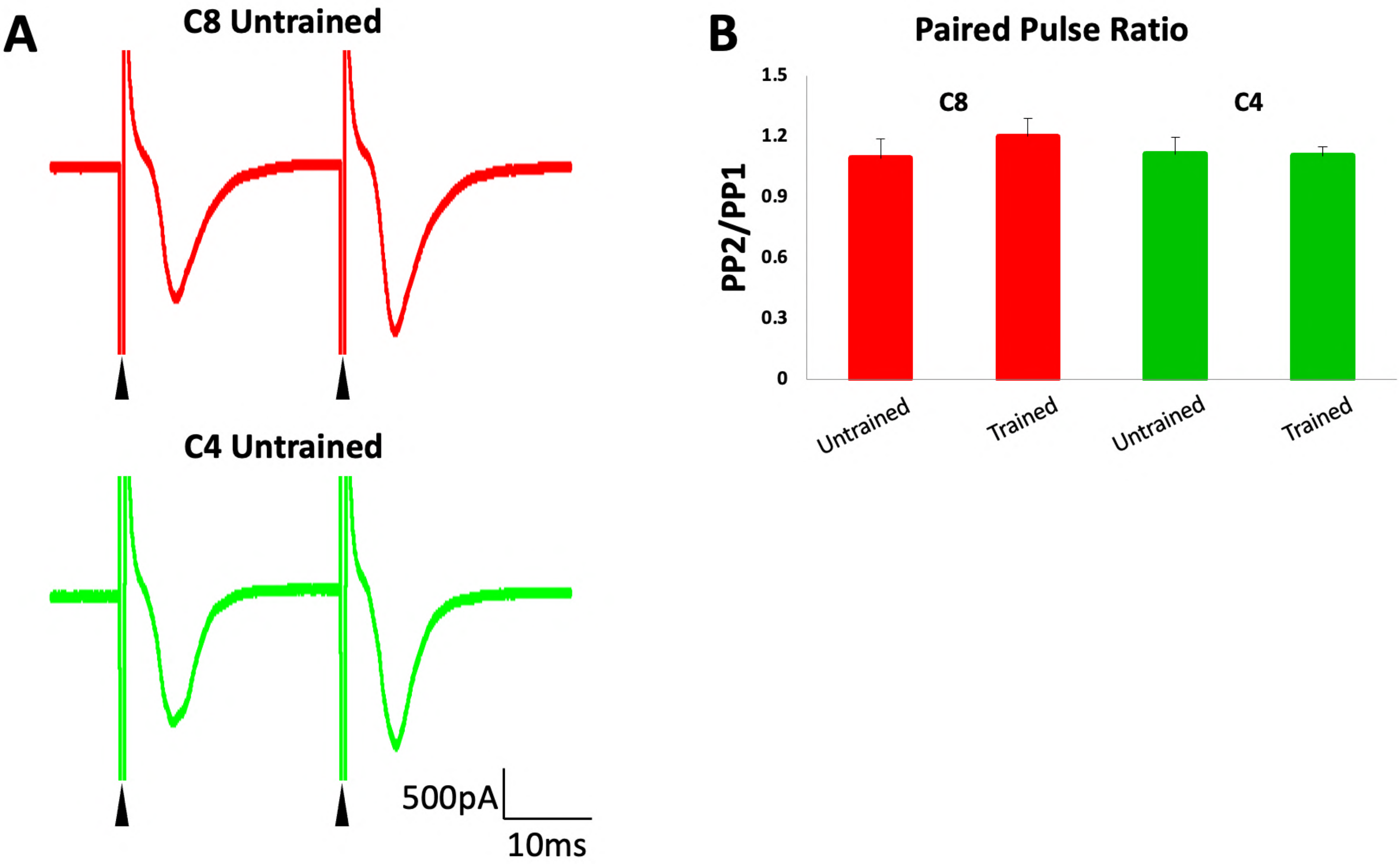

Supplemental Figure 6

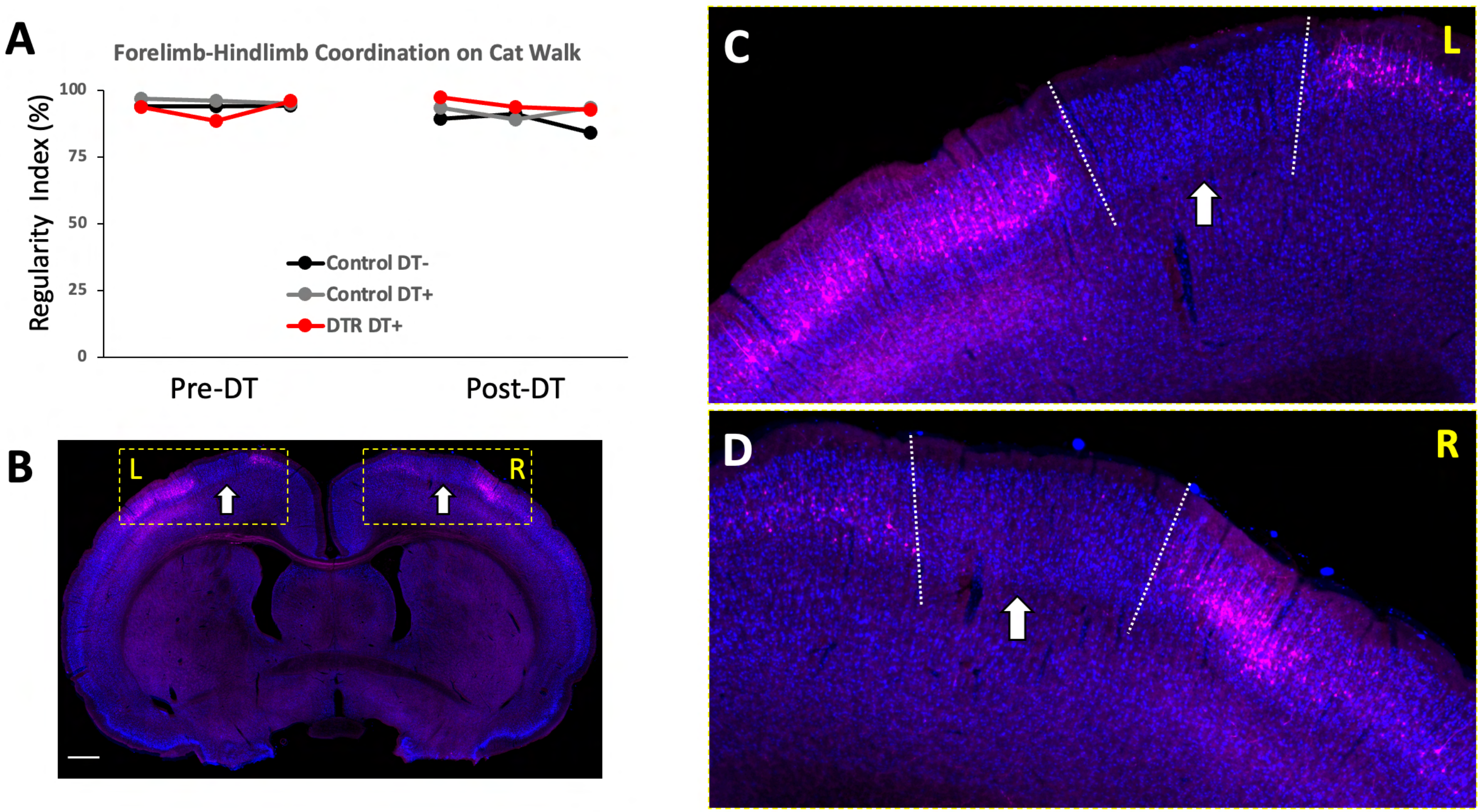

Supplement: 1 [file NIHMS1988737-supplement-1.pdf]
